# Impact of chronic painful temporomandibular disorders on quality of life

**DOI:** 10.22514/jofph.2024.017

**Published:** 2024-06-12

**Authors:** Bruno Macedo de Sousa, David Neves, Jose Antonio Blanco Rueda, Francisco Caramelo, Maria Joao Rodrigues, Nansi López-Valverde

**Affiliations:** Institute for Occlusion and Orofacial Pain Faculty of Medicine, University of Coimbra, 3004-504 Coimbra, Portugal; Department of Surgery, University of Salamanca, 37007 Salamanca, Spain; Biomedical Research Intitute of Salamanca (IBSAL), 37007 Salamanca, Spain; Laboratory of Biostatistics and Medical Informatics, Institute for Clinical and Biomedical Research (iCBR), School of Medicine, University of Coimbra, 3000-548 Coimbra, Portugal

**Keywords:** Anxiety, Biopsychosocial model, Daily activity, General mood, Oral health-related quality of life, Orofacial pain, Temporomandibular disorders

## Abstract

Temporomandibular Disorders (TMDs) are pathologies based on multifactorial etiology and a biopsychosocial model, where anxiety becomes one of the most important psychological factors as it is the most frequent symptom presented in most of the population at some point in life. Taking into account the need for a multidisciplinary approach, we set out to evaluate the possible impact of orofacial pain on patients’ quality of life. In this study, the sample population FROM the Medical School of the University of Coimbra (Portugal) was evaluated using two tools: Diagnostic Criteria for Temporomandibular Disorder (DC/TMD) and an adaptation of the West Haven-Yale Multidimensional Pain Inventory. Participants with Chronic Pain for more than three months and diagnosed with TMD were included in the research. The quality of life and pain intensity of participants with Chronic Orofacial Pain were assessed using questionnaires. Subsequently, statistical analysis were conducted. A total sample of 122 participants was selected. A statistically significant association was observed between an increase in pain intensity and a decrease in quality of life in three aspects we considered (daily activity, general mood and anxiety) and we demonstrated that pain intensity is significantly associated with a decrease in quality of life.

## 1. Introduction

The term “orofacial pain” (OP) consists of two terms: oral indicating pain inside the mouth and facial which includes pain below the orbitomeatal line, above the neck and in front of the ears [1]. It is associated with pain and dysfunction in the hard and soft structures of the head, neck and face, which send impulses through the trigeminal nerve, thus affecting motor and sensory transmission. The pathological range can go from headaches to neurogenic pathologies, vascular and even pain secondary to primary cancer or, even, inflammatory diseases such as pericoronitis [2]. In the universe of these conditions, the American Academy of Orofacial Pain (AAOP) defines temporomandibular disorders (TMDs) as a general term that applies to musculoskeletal and neuromuscular disorders, involving not only the masticatory muscles and temporomandibular joints (TMJ), but also adjacent structures, presenting as pathologies that may be associated with an orthopedic and functional imbalance of the cranio-cervical-condylar-mandibular complex [3].

TMDs represent a significant public health problem, and is considered the most common cause of chronic pain of non-dental origin in the orofacial region and the second most common musculoskeletal condition causing pain and disability (after chronic low back pain). Its prevalence is around 31% in adults and 11% in children and adolescents, affecting mostly females (in a 2 to 1 ratio) [4, 5]. Furthermore, this type of pathology is a costly process for both patients and health services since, in addition to medical intervention, it often requires multidisciplinary approaches and costly medication that is estimated to be higher than the annual cost of cancer or diabetes pathologies. A study carried out by Alisson *et al*. [6] in the UK reported direct costs of £333 and indirect costs of £1242, per person over a 6-month period.

The main symptoms of this type of pathology correspond to the presence of pain, limitation of mobility, mouth opening and joint noises. Pain in the articular and/or muscular regions is usually the main complaint, which may be intermittent or persistent, worsening with chewing, talking and yawning [4, 7]. The etiology of TMD is considered multifactorial and complex and is currently based on the biopsychosocial model, in which precipitating, predisposing and perpetuating factors are related to each other and are responsible for the cause, progression and increased risk of the onset of the pathology [8]. These factors, either synergistically, or as a single assimilating factor of the different functions, contribute to the alteration of the functional and orthopedic balance of the constituent structures of the stomatognathic system, playing an important role in the chronicity of pain and its impact on the patient’s quality of life [9, 10, 11].

Regarding the diagnosis of TMDs, the Diagnostic Criteria for Temporomandibular Disorder (DC-TMD) are currently recognized as an instrument agreed upon by the scientific community from the aforementioned multifactorial perspective. Divided into two axes, the evaluation of physical, psychological and psychosocial alterations allow patients to be included in different predefined groups and subgroups, ranging from muscular, articular and degenerative disorders to headaches attributed to TMDs [12].

Musculoskeletal disorders can have consequences for various aspects of the physical and mental well-being of patients. Moreover, chronic conditions, such as fibromyalgia and TMDs, are responsible for a negative impact on the quality of life. These types of pathology lead to various forms of psychological suffering such as anxiety, stress and depression, leading to reduced work capacity, economic difficulties associated with the constant need for health services, social phobias and sleep disorders [13].

Oral health-related quality of life (OHRQoL) is a recent concept, defined as a multidimensional approach that reflects a person’s comfort in eating, sleeping and interacting socially. It is also associated with self-esteem and satisfaction with one’s oral health, as well as functional, psychological, social factors and experiences of pain and discomfort [14, 15]. Durham *et al*. [16] were responsible for creating a measurement instrument that associates TMJ and masticatory muscle pathologies with OHRQoL, whose reliability, validity and discriminative ability have been established in several subsequent studies.

When assessing the biopsychosocial model, anxiety becomes one of the most important psychological factors to be considered. It is the most common symptom, occurring in most of the population at some point during their life. Although there is the possibility of it being pathological, depending on the intensity of the emotion, anxiety can be described as a universal experience of any human being, a normal emotional reaction with physiological and psychological components, facing various environmental situations. This response to a stimulus may result in some physical symptoms such as increased blood pressure, body tremors, headaches, insomnia, paresthesia in the extremities and a feeling of dyspnea, and psychological reactions such as panic attacks [17]. Anxiety can influence the pain threshold by altering the nociceptive impulses of the central nervous system and the release of neurotransmitters. As a result, patients with generalized anxiety disorders suffer much more frequently from chronic pain, as well as increased muscle tone, sleep disturbances, restlessness and concentration deficits. It can also lead to increased duration, frequency and intensity of parafunctional habits responsible for hyperactivity of the masticatory muscles, with consequent overloading of the TMJs [18, 19].

The aim of our study was to evaluate, from a multidisciplinary approach, the possible impact that orofacial pain may have on patients’ quality of life.

## 2. Materials and methods

The sample was recruited from consultations at the Integrated Clinical Unit of the Faculty of Medicine of the University of Coimbra.

The project used two assessment instruments: DC/TMD and the West Haven-Yale multidimensional pain inventory completed by the participants. Inclusion criteria for participants were defined as follows: ages 18 years or older and participants who were not pregnant. The exclusion criteria were edentulous patients and patients with orofacial pain not related with TMD or orofacial pain for less than 3 months.

All patients meeting the inclusion criteria who attended a dental appointment at the Coimbra Hospital and University Center (CHUC) Dental Clinic, were evaluated according to the DC-TMD, in order to diagnose the presence or absence of myalgia, myofascial pain or arthralgia and if they were present for longer than 3 months or not. Only patients with chronic pain related to TMD were presented with the objectives of the study and were asked to sign the informed consent form to be included in the research project. Subsequently, they were asked to complete the West Haven-Yale multidimensional pain inventory, a validated and reproducible instrument, developed to assess the impact of chronic pain on patients’ lives, the interaction between other people and patients when they communicate their pain status, as well as patients’ participation in daily activities [20]. In this study, a validated version that was translated and culturally adapted for the Portuguese population was used with adequate psychometric properties (https://www.aped-dor.org/socios/material_bibliografico/diversos_Questionarios_Dor-Rev_DOR_Volume15-n4-2007.pdf).

Subsequently, some questions and answers from the multidimensional pain inventory were selected to assess pain intensity in three dimensions of quality of life: activities of daily living (questions 3, 8, 17 and 19), general mood (questions 6, 9, 12, 13 and 14) and anxiety (questions 18 and 20). The pain intensity scale was defined as follows according to the answer to the question 1 of the questionnaire: no pain (score 0), mild pain (1 and 2), moderate pain (3 and 4) and severe pain (5 and 6).

The sample’s demographic details, including age and gender, were presented. Age statistics, such as the mean, standard deviation and percentiles (25th and 75th), were utilized. Gender distribution was explored through both absolute counts and relative frequencies.

The study explored the three dimensions regarding quality of life, scrutinizing disparities across three distinct OP groups—mild, moderate and severe. This comparative analysis was executed through a Multivariate Analysis of Variance (MANOVA). To validate the homogeneity of variances assumption, the Box’s M Test was applied. The significance of MANOVA outcomes was assessed *via* the Wilk’s Lambda value. Subsequently, each quality-of-life dimension underwent assessment through Analysis of Variance (ANOVA). This was accompanied by Tukey *post-hoc* tests, and elucidated further through the creation of confidence interval charts. Additionally, the study examined the influence of gender on quality-of-life facets. Boxplots depicted the distribution of values for activities of daily living, general mood and anxiety in male and female groups. Medians were compared using the Mann-Whitney test, and the normality assumption was tested with the Shapiro-Wilk test.

Statistical analysis was performed in the IBM® SPSS® v27 platform (Armonk, NY, USA) and MS® Excel® platform 2019 (16.0) (Redmond, WA, USA) with a significance level of 0.05.

## 3. Results

A sample of 122 participants was collected. In this group, 97 participants (79.5%) were female and 25 (20.5%) were male. The mean age was 42.9 years.

The MANOVA analysis revealed an overall significant difference between pain groups on the dependent variables of the quality of life (Wilks’ λ = 0.167, *F* (3, 117) = 194.3, *p *< 0.001). This indicates that, overall, there is a significant difference in multivariate means between groups. To investigate the sources of this difference, univariate analyzes were performed on each dependent variable.

Daily activities: Significant differences were found between groups, *F* (2, 122) = 13.8, *p *< 0.001. *Post hoc* tests indicate that the Mild group differs significantly from the Moderate group (*p* = 0.010) and from the Severe group (*p *< 0.001). However, no statistically significant differences were observed between Moderate and Severe groups (*p* = 0.068).

General mood: Significant differences were found between groups, *F* (2, 122) = 14.9, *p *< 0.001. *Post hoc* tests indicate that the Severe group differs significantly from the Mild group (*p *< 0.001) and from the Moderate group (*p* = 0.007). However, no statistically significant differences were observed between Mild and Moderate groups (*p* = 0.056).

Anxiety: Significant differences were found between groups, *F* (2, 122) = 11.5, *p *< 0.001. *Post hoc* tests indicate that Mild group differs significantly from the Moderate group (*p *< 0.001) and from the Severe group (*p* = 0.010). However, no statistically significant differences were observed between Moderate and Severe groups (*p* = 0.730).

All the statistical analysis between groups is summarized in Table 1.

The following graphs (Figs. 1,2,3) show the confidence intervals for each of the quality-of-life dimensions evaluated according to the levels of intensity of orofacial pain.

Gender exhibits statistical differences in terms of quality of life. Specifically, daily activities (*p *< 0.001), general mood (*p *< 0.001), and anxiety (*p* = 0.012) tend to demonstrate higher scores in female subjects with OP, as illustrated in the subsequent boxplots (Figs. 4,5,6).

**Table 1.** Statistical analysis of the quality-of-life survey scores in the dimensions of daily activity, general mood and anxiety for the three levels of pain intensity (mild, moderate and severe).

	Mild pain (83)	Moderate pain (26)	Severe pain (13)	*p*
Daily activities	1.91 (1.39) 0.80/3.00	2.81 (1.00) 2.00/3.60	3.83 (1.60) 2.60/4.80	<0.001^c^
General mood	2.12 (1.38) 1.00/3.00	2.79 (1.00) 2.25/3.25	4.13 (1.04) 3.00/4.50	<0.001^c^
Anxiety	2.83 (1.53) 2.00/4.50	4.08 (1.51) 3.00/5.50	4.46 (1.18) 4.50/5.00	<0.001^c^

*^c^: Kruskal-Wallis test; p: probability value.*

**Fig. 1.** Confidence intervals of the scores related to daily activities according to the levels of pain intensity. For each degree of pain intensity, the minimum and maximum values were identified. The average line shows the impact on quality of life as pain intensity increased.

**Fig. 2.** Confidence intervals of general mood scores according to the levels of pain intensity. For each degree of pain intensity, the minimum and maximum values were identified. The average line shows the impact on general mood as pain intensity increased.

**Fig. 3.** Confidence intervals of anxiety scores according to the levels of pain Intensity. For each degree of pain intensity, the minimum and maximum values were identified. The average line shows the impact on anxiety levels as pain intensity increased.

**Fig. 4.** Boxplot of daily activities scores categorized by gender. The line in the middle represents the median value, the whiskers are the minimum and maximum value, the star is the extreme value, and the white circle is the outlier. F: female; M: male.

**Fig. 5.** Boxplot of general mood scores categorized by gender. The line in the middle represents the median value, the whiskers are the minimum and maximum value, and the black circle the outlier. F: female; M: male.

**Fig. 6.** Boxplot of anxiety scores categorized by gender. The line in the middle is the median value. F: female; M: male.

## 4. Discussion

Our study was evaluated from a multidisciplinary perspective (relationship level and participation of the subjects in daily activities), the possible impact that Chronic Pain of temporomandibular disorders could have on the quality of life of the patients and showed that pain intensity is significantly associated with a decrease in quality of life of the patients included.

Currently, according to evidence based on the biopsychosocial model, it would be wise to suggest that TMDs, especially in chronic cases, impair and reduce the quality of life of the patients suffering from them. In fact, psychological factors such as anxiety, stress and depression are predominant in the alteration of pain threshold and nociceptive impulses of the Central Nervous System [21, 22] and in our study, quality of life was assessed under the dimensions of general mood, impact on the performance of daily activities and changes in anxiety levels.

Aranha *et al*. [23] conducted a systematic review of the association between job stress and TMDs in adult paid workers and, despite not finding conclusive results, reported that 50% of the studies consulted, found a positive association between stress and TMDs diagnosis in several job categories; however, authors such as Han *et al*. [24] in a study carried out among a large female sample, found a significant association between working hours and TMDs, and reported that there is a higher risk of developing TMDs when the population works more than 60 hours per week, despite not finding a significant association between TMDs and satisfaction with shift work, or temporary work. Our study found that pain could influence work activity and work capacity, impacting both parameters, agreeing with others who reported that job dissatisfaction could lead to absenteeism [25]. The prevalence of oral dysfunction has been associated primarily with reduced worker performance and productivity (absenteeism) rather than lost workdays (presenteeism). A study on absenteeism and presenteeism related to oral health reported that 28–50% of absenteeism cases were caused by oral problems, with toothache and temporomandibular joint pain being the most frequent reasons [26]. Chronic pain associated to Temporomandibular Disorders can be imperceptible or even accommodating for the working class, delaying and making it difficult to seek help, which is aggravated by underdiagnosis of musculoskeletal pathologies. Svebak *et al*. [27] demonstrated in a large sample of more than 30,000 employees, that the intensity of chronic pain has a great impact on dissatisfaction and work capacity, promoting a recurrent absence of the patient, or the presence of weak physical/psychological conditions, both in their work activity, as well as in daily activities.

The OPPERA (Orofacial Pain Prospective Evaluation and Risk Assessment) study was revolutionary and essential to determine the psychological, genetic and environmental risk factors, among others, that contribute to the development of TMD chronicity, however, it did not explore the testimonies of patients living real and routine situations, such as interpersonal relationships [28, 29, 30]. We also observed that patients with severe chronic pain presented more distress and general moodiness than those with mild pain. In this aspect Maracci *et al*. [31] in a cross-sectional study on a sample of 310 individuals, found a positive association between chronic pain associated with TMDs and the marital status of the patients included in the study, with patients with a partner being more likely to present chronic pain with high disability, than those who were single or divorced/widowed; however, other studies reported that daily coexistence with pain was more bearable in patients who were satisfied with their partner relationship than in those who had an unsatisfied relationship [32]. Concerning anxiety, taking into account the questions related to irritability and tension/anxiety to which the patient was subjected, we found statistically significant differences, comparing the different levels of pain intensity. Our study, compared with others that used specific anxiety assessment tools, alone or associated with other psychosocial domains, such as the Oral Health-Related Quality of Life (OHRQoL), found a positive correlation between anxiety levels and chronic pain intensity in patients with TMDs [33, 34]. In the same way, different systematic reviews have highlighted the positive association between anxiety and TMDs [35, 36].

The hypothesis that psychological factors may contribute to TMDs was developed in the 1970s and since then, much attention has been paid to the influence of emotional traits in TMDs [37, 38]. Ferrando *et al*. [39] observed that patients suffering from TMDs are more prone to stress and are more likely to suffer from emotional stress. A study by Yap *et al*. [40] of 455 subjects, observed that those with TMD showed substantially higher somatization and psychological distress scores than those without. A large OPPERA cohort study of 2737 subjects with a follow-up of 5.2 years, identified psychological factors as predisposing risk factors for the development of pain in TMD and that, in general, these psychological influences are similar across demographic categories [28].

Therefore, a psychometric instrument called the Minnesota Multiphasic Personality Inventory (MMPI) was designed to differentiate between psychological and physical causes of chronic pain [41]. A recent study conducted by Hekmati *et al*. [42] applied this modified tool (MMPI-2-RF, Minnesota Multiphasic Personality In-ventory-2-Reconstructed) to evaluate a sample of 258 subjects and found significant differences between the means of state/trait anxiety in patients with TMDs and controls. The association of these data could be explained by the fact that psychological factors are enhancers of parafunctional habits, lowering the pain threshold and altering the sensitivity of the masticatory muscles [18].

Chronic pain is a risk factor for anxiety and depression and suicides have been described in patients with both pathologies. However, patients with depression, tend to have higher levels of pain compared to those who are not depressed, and it has been shown through combined diagnostic methods, that decreased levels of depression are associated with decreased pain intensity [43]. Pincus and Morley suggested that, in the processing of information in chronic pain, the biases that appear would be the result of the superimposition of 3 schemas: pain, disease and those inherent to the individual, and precisely, the pain and disease schemas, could increase distress and the evolution of the disease in patients with chronic pain [44]. In line with this study, and despite these limitations and biases, we found in our study that patients with severe and chronic painful Temporomandibular disorder had higher levels of distress than patients with moderate levels of pain.

Moreover, it is known that both sex (biological construct) and gender (social construct) intervene in the response to pain. Some studies have highlighted the differences in the response to pain between men and women, with women presenting greater prevalence and manifestation than men [45] and it has even been described that men with more masculine traits manifest higher pain thresholds than those with feminine traits [46]. In agreement with these reports, our investigation found significant statistical differences, especially in the performance of daily activities and general mood (*p *< 0.001), in female subjects with chronic painful temporomandibular disorders.

## 5. Conclusions

An increase in the intensity of chronic painful temporomandibular disorders is associated with a decrease in quality of life. Therefore, it is essential that this pain be considered in psychosocial aspects, such as work and daily activities, always taking into account the possible repercussions for patients who suffer from it.
